# Enhanced Efficiency of Carbon-Based Mesoscopic Perovskite Solar Cells through a Tungsten Oxide Nanoparticle Additive in the Carbon Electrode

**DOI:** 10.1038/s41598-019-45374-x

**Published:** 2019-06-19

**Authors:** Lin Zhou, Yuhua Zuo, Tapas Kumar Mallick, Senthilarasu Sundaram

**Affiliations:** 1 State Key Laboratory on Integrated Optoelectronics, Institute of Semiconductors, Chinese Academy of Sciences, Beijing, 100083 China; 2 Center of Materials Science and Optoelectronics Engineering, University of Chinese Academy of Sciences, Beijing, 100049 China; 3 Renewable Energy, Environment and Sustainability Institute, University of Exeter, Cornwall, TR10 9EZ UK

**Keywords:** Solar cells, Nanoparticles

## Abstract

This paper presents perovskite solar cells employed with WO_3_ nanoparticles embedded carbon top electrode. WO_3_ nanoparticles works as an inorganic hole-transport material (HTM) to promote the hole-extraction in the perovskite/carbon interface as revealed by efficiency, electrochemical impedance and external quantum efficiency measurements. As a result, a 40% enhancement of energy conversion efficiency has been achieved compared to the reference devices with the energy conversion efficiency of 10.77% under standard conditions. In addition, the Li-TFSI can modify the interface between electron-transport material (ETM) and perovskite, which may inhibit the recombination at the ETM/perovskite interface. The V_OC_ of devices upon the modification of Li-TFSI is increased from 887.9 to 934.2 mV. This work highlights about the enlightenment of the effective performance of carbon-based mesoscopic PSCs by the introduction of HTM and the modification of interfaces.

## Introduction

The world is now front of a severe status to find new strategies for cheap and clean energy alternatives. Solar energy conversion is one solution to meet the world’s energy needs. Perovskite solar cells (PSCs) based on lead halide perovskite (e.g., CH_3_NH_3_PbI_3_) have recently attracted attention in the photovoltaic industry because of their low costs and high efficiency^1–6^. The power conversion efficiencies (PCEs) of these devices have increased from ~4.0% to 23.3%^1,7^. Mostly, best performing PSCs are based on evaporated costly metallic back electrodes such as Ag and Au due to their superior conductivity and reflectivity. To using such costly metal electrodes require energy-intensive vacuum-evaporation techniques and disrupt at ambient condition. Furthermore, Ag exhibits strong reactivity with the halogens^8^ and Au could interfuse into the perovskite layer causing device degradation^9–11^, which will further suppress their long-term use and large scale commercialization.

Carbon as an alternative abundant source explicit a cheaper and facile way to replace the cost-effective metal back electrodes in PSC. The carbon and their composite exhibits chemical resistant towards oxidation/reactions and can be printed in batch or continuous roll-to-roll processing^12–14^. Han and colleagues explored carbon electrodes in hole-transport material-free (HTM-free) PSCs with a TiO_2_/ZrO_2_/C configuration and obtained an efficiency of 6.64% of power conversion (PCE)^15^. A series of works with such carbon-based electrode has been already reported by various reseachers^16–20^. Though, most researches focused on the crystallization of perovskite and the efficiency of HTM-free PSCs using carbon electrode has reached up to 15%^12,21^. Moreover, the efforts to improve the performance of HTM-free carbon-based PSCs have been extended to a much wider range, such as electron-transporting material (ETM) and its interface^22,23^, the composition of the carbon electrode itself^15,16,24,25^, and even the reintroduction of p-type materials in HTM-free PSCs^20,26,27^.

Since the cost of organic HTM, such as spiro-OMeTAD [2,2′,7,7′-tetrakis(N,N-di-p-methoxyphenyl-amine)9,9′-spiro-bifluorene] polymer-based poly(triarylamine)(PTAA), is extremely high for large-scale applications, and the organic HTMs or their ingredients are clearly a main factor in the long-term operational and thermal instability of the PSCs with them^28^, the HTM-free PSCs have become an attractive option. However, the “HTM-free” approach is not the only pathway to combat these issues of cost and instability, and the carbon-based mesoscopic PSCs should not be restricted to “HTM-free”. The inexpensive inorganic hole extraction layers, such as CuSCN^29,30^, NiO^20,31–33^, could also be used in carbon-based mesoscopic PSCs to conquer these issues of organic HTM. Recently, NiO nanoparticles as an inorganic HTM has been introduced in the carbon-based PSCs which resulted a highest efficiency of 17%^26,27,34^. Although the reintroduction of HTM (NiO) in carbon-based PSC device structure was proposed a few years ago, the alternatives are rarely covered in the photovoltaic field.

In this study, we introduce the hole-extraction material tungsten oxide (WO_3_)^35–38^ into the carbon paste used for the fabrication of the counter electrode to improve the efficiency of carbon-based PSCs. In addition, we used the lithium bis(trifluoromethanesulfonyl)imide (Li-TFSI) to modify the mp-TiO_2_ layer’s surface, which could greatly affect the open-circuit voltage (Voc) of cells, as the Li-TFSI can modify the interface between ETM and perovskite, which may decrease the number of deep traps, which act as recombination centers and suppress the recombination at ETM/perovskite interface, improving the open circuit voltage^39^. As a result, with the introduction of the additive WO_3_ nanoparticles and modification of Li-TFSI, the average PCE of carbon-based PSCs was increased from 7% to 10%, where the V_OC_ raised from 886 to 931 mV particularly, and a highest PCE of 10.8% was realized. It indicates there are much potential to further cultivate the performance of carbon-based mesoscopic PSCs by the introduction of HTM followed by the interface modification

## Results and Discussion

**Figure 1:**
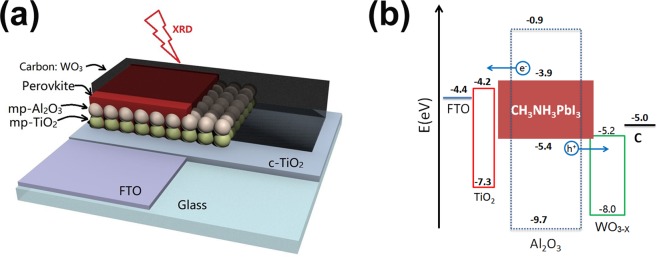
Schematic structure (**a**) and energy band diagram (**b**) of carbon-based mesoscopic PSCs with WO_3_ nanoparticles additive. Values for WO_3_ was measured using UPS (Fig. S2, Supporting Information).

The whole schematic structure of carbon-based mesoscopic PSCs is shown in Fig. 1a. The films of mesoporous TiO_2_ and Al_2_O_3_ are spin-coating on a Glass/FTO/c-TiO_2_ substrate layer by layer. WO_3_ nanoparticles are embedded in carbon layer as an additive. Then, perovskite precursor is seeped into the mesoporous layers directly with drop-casting process, and spin-coating at 1000 rpm for 15 s to form flat films and avoid the uncertainties of human hands (Fig. S1, Supporting Information). Figure 1b shows a simple energy band schematic diagram of the working principle of the carbon-based mesoscopic PSCs with WO_3_ nanoparticles additive. Based on the energy level positions of the device components, the excited electron is transferred from the conduction band (CB) of the perovskite layer (−3.9 eV) to that of TiO_2_ layer (−4.0 eV) followed by the hole extraction from perovskite layer (−5.4 eV) to carbon layer (−5.0 eV) via WO_3_ (−5.3 eV)^36,37^. The ETM and carbon electrode are separated by the mesoporous Al_2_O_3_ film served as spacer which retard the electron-hole recombination in the PSCs. The additive WO_3_ inside carbon film can work as HTM to promote the hole-extraction in perovskite/carbon interface due to its appropriate position of conduction band as shown in Fig. 1b.

**Figure 2:**
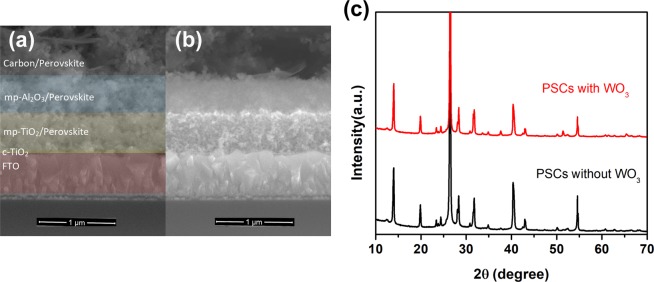
SEM cross-sectional images of PSCs showing device architectures without (**a**) or with (**b**) WO_3_ nanoparticles additive. (**c**) X-ray diffraction (XRD) patterns of devices deposited on mesoporous FTO/TiO_2_/Al_2_O_3_/C substrates with or without WO_3_ nanoparticles. Figure 1a shows the directions for the XRD (from the carbon side) measurements.

The cross-sectional SEM images of the mesoporous structure of the FTO/c-TiO_2_/mp-TiO_2_/mp-Al_2_O_3_/C composite with or without WO_3_ additive are shown in Figs 2a,b and S3. The additive WO_3_ nanoparticles existing in mesoporous carbon layer have rarely effect on the structure of devices. Here, the thicknesses of mp-TiO_2_ and mp-Al_2_O_3_ are ~600 and ~500 nm^25^, which are optimized by series of controlled trials, respectively. For the carbon-based mesoscopic PSCs, the XRD patterns (Fig. 2c) of our devices are consistent with previous reports^12,13,22^. The XRD spectra of devices with or without WO_3_ display no differences, indicating that the traces of additive WO_3_ nanoparticles had little effect on the crystallization of perovskite inside. Besides, the introduction of WO_3_ will not affect the absorption of device (Fig. S4). However, the existence of WO_3_ could be proved by the energy dispersive X-ray spectrum (EDS) characterization in Fig. S3. In short, the introduction of traces of additive WO_3_ nanoparticles did not affect the structure, morphology of device and crystallization of perovskite inside.

**Figure 3:**
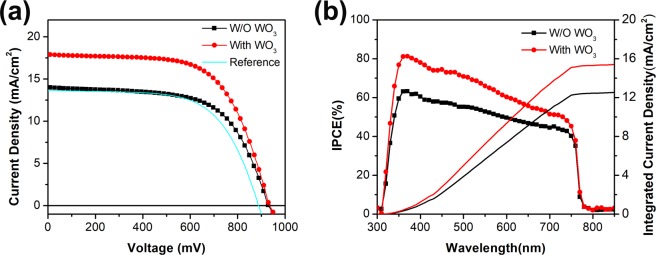
(**a**) Current–voltage (*J–V*) characteristics of PSCs with (With WO_3_, red circle) or without (W/O WO_3_, black square) WO_3_ additive and the reference (without WO_3_ and without Li-TFSI, cyan line) under simulated AM 1.5 (100 mW/cm^2^) at room temperature. (**b**) The IPCE spectra and integrated current density of reference and PSCs with WO_3_ nanoparticles additive.

Figure 3a shows the current density–voltage (*J–V*) curves of PSCs with or without WO_3_ additive measured under simulated AM 1.5 (100 mW/cm^2^). As shown in Table 1, the reference PSCs exhibited the highest PCE of 7.67% with a V_OC_ of 887.9 mV. The Li-TFSI modified PSCs with WO_3_ additive exhibits an increased PCE of 10.77% with a J_SC_ of 17.96 mA/cm^2^ and a V_OC_ of 934.2 mV. Figure 3b indicates the IPCE spectra of the PSCs in the range from 300 nm to 850 nm. The WO_3_ additive carbon based PSCs exhibits higher IPCE value compared to the without WO_3_ additive device. A promotional hole extraction property of WO_3_ additive is believed to contribute significantly to the increased photo-generated current, leads to enhance the efficiency. The integrated photocurrents calculated from the overlap integral of the IPCE spectra with the AM 1.5 solar emission are also shown in Fig. 3b. The integrated photocurrent of the PSCs with WO_3_ additive is 15.8 mA/cm^2^, which agrees closely with the photocurrent density of 16.2 mA/cm^2^ measured at the beginning of testing, which rose up to 17.96 mA/cm^2^ after 5~10 s of light soaking. In addition, both devices show terrible hysteresis, as shown in Fig. S5 (Supporting Information). Moreover, several reports are on carbon-based mesoscopic PSCs mostly based on varied configurations and MAPbI_3_. Some of them exhibit good performances with regard to short-circuit current density, open-circuit voltage, fill factor, efficiency and active area as shown in Table S1.

**Table 1:** Device performance of PSCs (active area 0.30 cm^2^) with or without WO_3_ additive under simulated AM 1.5 (100 mW/cm^2^) conditions.

Device	Jsc (mA/cm^2^)	Voc (mV)	FF	Efficiency
Reference	13.68	887.9	63.1%	7.67%
W/O WO_3_	13.98	929.8	62.4%	8.11%
With WO3	17.96	934.2	64.2%	10.77%

**Figure 4:**
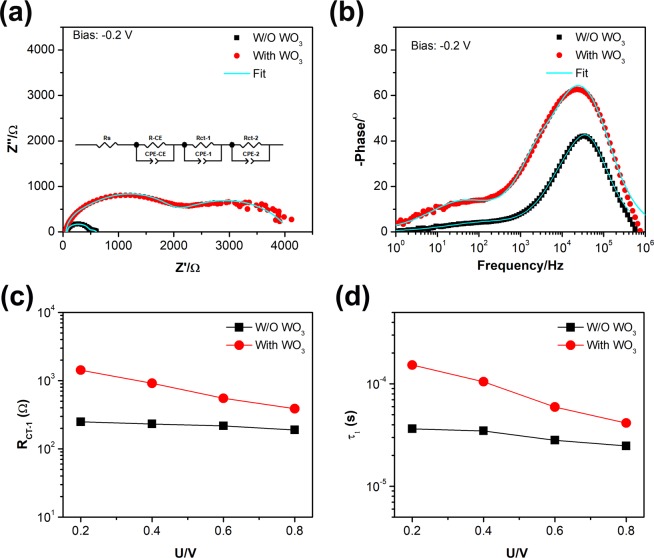
Electronic impedance spectroscopy (EIS) characteristics of PSCs with (With WO_3_, red circle) or without (W/O WO_3_, black square) WO_3_ additive. Electrochemical impedance spectra in the form of Nyquist plots (**a**, left) and Bode phase plots (**b**, right) measured under dark with bias at −0.20 V. Inset: the equivalent circuit for fitting the EIS data. The solid cyan lines are the fitted curves. Plots of charge recombination resistances (**c**, R_CT-1_) and lifetimes (d, τ_1_) at the WO_3_/perovskite interface obtained from impedance measurements under no extra illumination at the given bias.

To understand the device electrical properties the electrochemical impedance spectroscopy (EIS) measurements were conducted for the PSC devices. Figures 4a,b and S6 show the Nyquist plots and the corresponding Bode phase plots in the frequency range from 1 MHz to 1 Hz for devices with or without WO_3_ additive under dark at a bias of −0.8, −0.6, −0.4 and −0.2 V, respectively. The semicircle at high frequency region (10^4^ to 10^6^ Hz) was attributed to the charge transfer resistance at carbon/perovskite interface (R_CE_)^25,40^. Here, we used three R_C_-circuit in series as an equivalent circuit, as shown in inset of Fig. 4a, to fit the impedance data. The obtained charge recombination resistances and capacitances for the two devices are summarized in Table S2, which shows R_S_ of PSCs with WO_3_ is less than the device without WO_3_, which suggests that the hole extraction process at the carbon electrode with WO_3_ is more efficient. A higher charge exchange resistances (R_CE_) at a bias of −0.80 V was observed for WO_3_ additive PSCs. compared to without additive device.

In case of without WO_3_ additive device, MAPbI_3_ acts as the light-harvester and hole transporter simultaneously. However, the p-type WO_3_ additive device may play the role of a hole collector along with the appropriate energy level to MAPbI_3_^27^. This may further enhance the hole extraction efficiency. Figure 4c,d present the obtained interfacial charge recombination resistance (R_CT-1_) and charge recombination lifetimes (τ_1_) at the WO_3_/perovskite interface for the device with WO_3_ or at the “p-type” MAPbI_3_/perovskite interface for the device without WO_3_ as a function of the applied bias. Figure 4c,d indicate that the device with WO_3_ exhibits larger charge recombination resistance (R_CT-1_) and longer larger charge recombination lifetime (τ_1_), which imply that WO_3_ nanoparticles could passivate the perovskite/carbon interface and inhibit the charge recombination^26^. The low frequency resistance region of EIS, corresponding to ion motion in the perovskite, is shown in Fig. S7. The small differences in τ_2_ between the two devices suggest that the photovoltaic performance of device would not be influenced by this element. In short, Figs 4a and S7 indicate that the WO_3_ nanoparticles with the degenerate energy level to perovskite could enhance the charge collection efficiency, which could be responsible for the improvement performance of the device.

**Figure 5:**
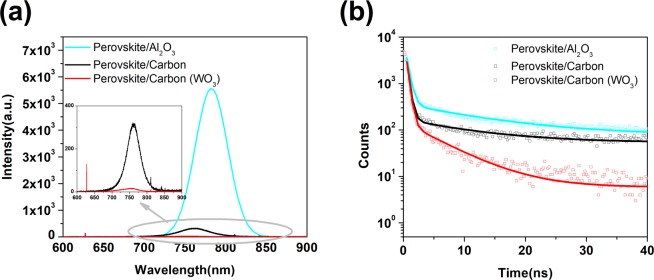
Steady-state photoluminescence (PL) spectra (**a**) and time-resolved PL (TRPL) decays (**b**) of perovskite (MAPbI_3_) on glass (black line), carbon electrode without WO_3_ (red line) and with WO_3_ (dark cyan line) films. Inset of (**a**): corresponding partially enlarged details. Excitation wavelength: 480 nm.

As discussed above, the hole extraction process at the carbon electrode with WO_3_ is more efficient than that without WO_3_ additive and the additive WO_3_ inside carbon film can work as HTM to promote the hole-extraction in perovskite/carbon interface. These could be also confirmed by the quenched steady state PL of the perovskite films, as shown in Fig. 5a. Figure 5a showed steady-state PL spectra of perovskite on the insulating glass and two kinds of carbon films with or without WO_3_, respectively. The steady-state PL of perovskite film was quenched when perovskite was formed in carbon films, which originates from fast charge transfer from the perovskite to carbon. The PL intensity of the carbon film with WO_3_ is much reduced compared with that without WO_3_ (inset of Fig. 5a), indicating that the hole extraction ability from the perovskite in the carbon electrode with WO_3_ is better than that without WO_3_ additive. In addition, Time-resolved photoluminescence (TRPL) decay measurements were performed to further evaluate the charge transfer at the perovskite/carbon interface with or without WO_3_. The result is shown Fig. 5b. The normalized TRPL decay shows that both perovskite/carbon and perovskite/carbon (WO_3_) samples exhibit faster quenching relative to the perovskite/Al_2_O_3_ sample, indicating a fast charge transfer from perovskite to carbon electrode after photon excitation. Moreover, the perovskite/carbon (WO_3_) exhibits a average lifetime (τ_ave_) of 1.25 ns, which is shorter than the PL lifetime of (2.89 ns), indicating that the WO_3_ nanoparticle additive could be acting as an effective additive to enhance hole extraction efficiency. This result could be originated from the appropriate position of conduction band of WO_3_ nanoparticles. The results of EIS, PL and TRPL spectra revealed that the carbon electrode with WO_3_ exhibited a more efficient charge transport and interfacial transfer than that without WO_3_ additive.

**Figure 6:**
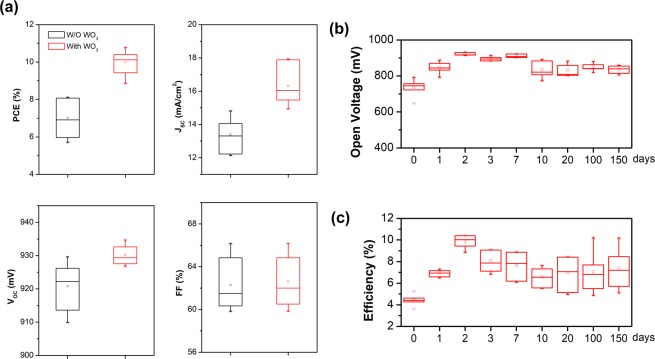
(**a**) Photovoltaic characteristics for 10 randomly selected PSCs devices with or without WO_3_ additive. (**b**,**c**) Stability data of a batch of PSCs with WO_3_ additive (10 cells) up to 150 days at ambient condition.

Figure 6a summarizes the statistical data relating to the numerical distribution of the key photovoltaic parameters for PSCs with and without WO_3_ additive. After the introduction of WO_3_, the average PCE was increased from 6.22 to 9.86%. It may be originated from the better electrical contact with WO_3_ additive at the perovskite/carbon electrode interface, which contributes to the hole extraction from perovskite. Based on aforementioned effects, the employment of WO_3_ nanoparticles as efficient additive in carbon electrode facilitating hole extraction. Stability of PSCs devices become a significant issue for their practical implantation. Figure 6b,c shows that the performance of devices will reach the best two days later and have long-term stability for over 3600 hours, which may be due to incomplete crystallization at the beginning for one-step growth. However, the PCE of the unsealed devices degenerate after reaching the best. However, the PCE exhibits decay to about 77% of the initial value within 100 hours. This results also indicates workout in aerobic condition is very sensible for PSCs mainly due to the degradation of perovskite layer and this issue will need to be solve for long –term practical implementation of PSCs.

## Conclusions

In summary, we demonstrated carbon electrode based perovskite solar cells (PSCs) utilizing WO_3_ nanoparticles with an appropriate position of conduction band as an additive in the carbon electrode to promote the hole-extraction in perovskite/carbon interface. The WO_3_ additive provides superior charge collection efficiency, achieving a cell with energy conversion efficiency of 10.77% in conditions of AM 1.5 G, which is a 40% increase of energy conversion efficiency compared to the reference devices. To the best of our knowledge, this is the first report of carbon electrode additive in carbon-based PSCs. We suggest that an efficient additive in the carbon electrode mesoporous structure has great potential to further enhance the photovoltaic performance of carbon-based PSCs.

## Materials and Methods

### Carbon paste was prepared as follows

10 g of graphite powder (Sigma–Aldrich) was mixed with 2 g of carbon black powder (Alfa Aesar) in 35 mL of terpineol (Sigma–Aldrich). Then, 1 g of ZrO2 powder (Sigma–Aldrich) and 15 g of ethyl cellulose (15 wt.% in ethanol) (Sigma–Aldrich) were added, followed by ball milling overnight. The additive was added before ball milling by 4 mL of WO_3_ nanoparticle ink (2.5 wt. % in isopropanol, Sigma–Aldrich, 793353).

### Perovskite precursor solution was prepared as follows

0.198 g CH_3_NH_3_I (MAI) (Sigma–Aldrich) and 0.573 g PbI_2_ (Sigma–Aldrich) were dissolved in 1 ml γ-butyrolactone (Sigma–Aldrich) and then stirred at 60 °C overnight.

### The fabrication of perovskite based mesoscopic solar cells

Fluorine-doped tin oxide (FTO) coated glass was first etched with HCl solution and zinc powder and then cleaned sequentially with detergent, deionized water, acetone, ethanol and deionized water. TiO_2_ compact layer was spin coated onto cleaned FTO transparent glass substrates by using 0.15 M titanium diisopropoxidebis-(acetylacetonate) (TAA) (75 wt% in isopropanol, Sigma–Aldrich) in a 2-propanol (99.9%, Sigma–Aldrich) solution at 2000 rpm for 30 s, followed by drying at 115 °C for 5 min and cooled down to room temperature. Further two successive coatings (2000 rpm, 30 s) from 0.3 M TAA solution were carried out and dried at 115 °C for 5 min. The dried TiO_x_ coated samples were heated at 415 ± 10 °C for 30 min in a hot plate. The mesoporous TiO_2_ layer was prepared with diluted TiO_2_ paste (18NRT from Dyesol; w/w = 1:3.5 in ethanol) by spin coating at 3000 rpm for 30 s and heated at 500 °C for 60 min. Further the mesoporous TiO_2_ layer were doped with lithium via spin coating (3500 rpm, 15 s) of 0.1 M lithium bis(trifluoromethanesulfonyl)imide (Li-TFSI) solution in acetonitrile and annealed at 415 ± 15 °C for 30 min. Then, the Al_2_O_3_ mesoporous layer was followed by spin coating with diluted Al_2_O_3_ paste (Sigma Aldrich, 702129; v/v = 1:2 in isopropanol) at 2000 rpm for 30 s and heated at 150 °C for 30 min. The mesoporous carbon layer was finally screen-printed with the as-prepared carbon paste and sintered at 450 °C for 30 min. After cooling down to room temperature, the perovskite precursor solution with an appropriate amount was infiltrated by drop casting via the top of the carbon counter electrode. After infiltrating active area of device, the device was spin-coating at 1000 rpm for 15 s. Finally, after drying at 50 °C for one hour, the mesoscopic solar cells containing perovskite were obtained.

### Characterization

The cross-section images of devices were imaged by a field-emission scanning electron microscope (SEM) (FEI Nova NanoSEM450) and the elemental microanalysis was measured with an Oxford Instrument X-MAXN EDS detector. The XRD spectra were obtained by a Bruker D8 Advance X-ray diffractometer with Cu Kα radiation (λ = 1.5418Å). A Solar Simulator (WXS-210S-20, AM1.5, Wacom Electric Co., Ltd.) was used to give an irradiance of 100 mW/cm^2^. The current-voltage characteristics of the cells with metal mask under these conditions were obtained by applying external potential bias to the cell and measuring the generated photocurrent with an *I-V* Curve Tracer (EKO MP-160, EKO INSTRUMENTS). The normalized incident-photon-to-electron conversion efficiencies (IPCEs) were measured using a PVE-300 Photovoltaic Characterization (BENTHAM INSTRUMENTS Ltd.). The impedance spectroscopy (IS) characterization of the devices was performed using an auto potentiostat/galvanostat instruments(Metrohm AutoLab B.V., NOVA) with the measured frequency range from 1 MHz to 1 Hz under the dark. Photoluminescence (PL) spectra and time-resolved PL decay spectra were measured using the Edinburgh Instruments (EI) FLS980 lifetime and steady state spectrometer.

## Supplementary information

Supporting information

## Data Availability

The datasets generated during and/or analysed during the current study are available from the corresponding author on reasonable request.
