# Supplementary material for: Enhanced Efficiency of Carbon-Based Mesoscopic Perovskite Solar Cells through a Tungsten Oxide Nanoparticle Additive in the Carbon Electrode
Source: Sci Rep. 2019 Jun 19;9:8778. doi: 10.1038/s41598-019-45374-x (PMC6584654; doi:10.1038/s41598-019-45374-x)
Supplement: Supplementary file 1 — Supporting information [file 41598_2019_45374_MOESM1_ESM.pdf]

Supporting Information

Enhanced Efficiency of Carbon-Based Mesoscopic Perovskite Solar Cells through a Tungsten Oxide Nanoparticle Additive in the Carbon Electrode

Lin Zhou[1,2], Yuhua Zuo[1,2*], Tapas Kumar Mallick[3], Senthilarasu Sundaram [3*]

1. State Key Laboratory on Integrated Optoelectronics, Institute of Semiconductors, Chinese Academy of Sciences, Beijing 100083, China.
2. Center of Materials Science and Optoelectronics Engineering, University of Chinese Academy of Sciences, Beijing 100049, China.
3. Renewable Energy, Environment and Sustainability Institute, University of Exeter, Cornwall TR10 9EZ, UK.

Corresponding Author:
*E-mail: yhzuo@semi.ac.cn *E-mail: s.sundaram@exeter.ac.uk

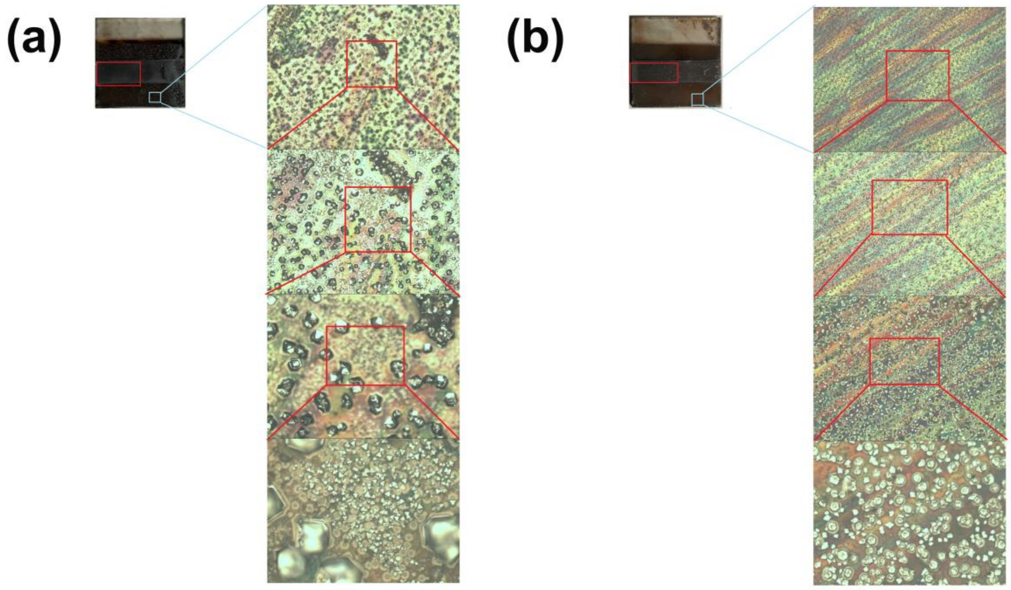

Figure S1. The pictures and corresponding optical microscope images of PSCs fabricated by only drop-casting (a) and combination of drop-casting and spin-coating (b) perovskite precursor.

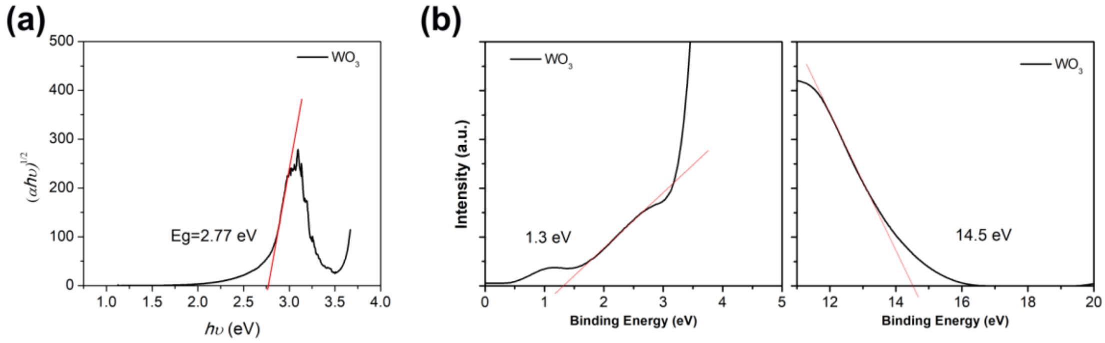

Figure S2. (a) Plots of $(\alpha h\nu)^{1/2}$ vs photon energy (eV) for WO_3 from UV-vis absorption spectra. (b) Ultraviolet photoelectron spectra (UPS) of the inelastic cutoff region and the onset region of WO_3.

As shown in Figure S2a, the optical band gap (Eg) of WO_3 is 2.8 eV.

As shown in Figure S2b, Valence band (VB) is – (21.2-(14.5-1.3)) eV=-8 eV.

Conduction band (CB)= VB+Eg= 5.2 eV.

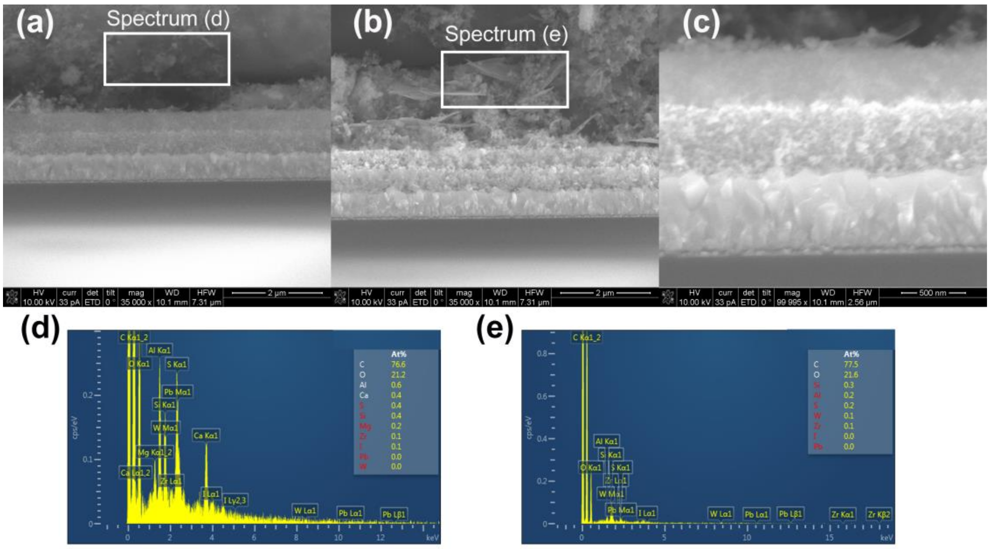

Figure S3. SEM cross-sectional images of PSCs showing device architectures without (a) or with WO_3 nanoparticles additive and the enlarged SEM cross-sectional image of Figure 2b. The white rectangles inside (a) and (b) are the measurement areas of energy dispersive X-ray spectrum (EDS) characterization. And the results are shown in (d) and (e), respectively.

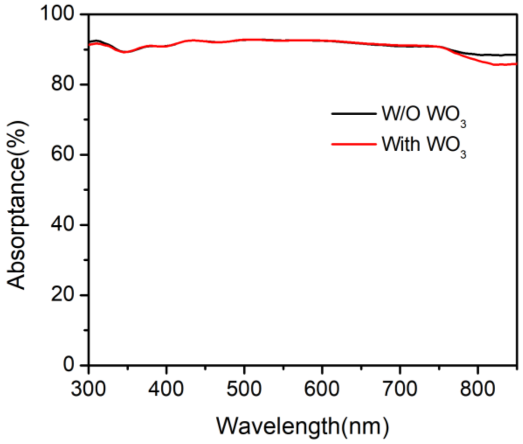

Figure S4. The absorption spectrum of PSCs with or without WO_3 additive.

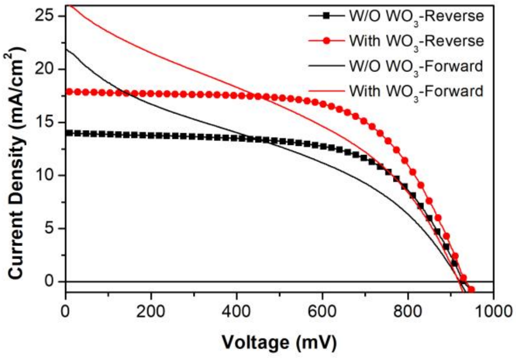

Figure S5. Current–voltage (J–V) characteristics of PSCs with (With WO_3, red) or without (W/O WO_3, black) WO_3 additive under simulated AM 1.5 (100 mW/cm^2) at room temperature. Line+symbols indicate the V_{OC}-J_{SC} measurement; Lines represent J_{SC}-V_{OC} measurement.

Table S1. Performance Comparisons of carbon-based mesoscopic perovskite solar cells from previous reports based on $MAPbI_3$.

Configuration	J_{SC} (mA/cm^2)	V_{OC} (mV)	FF	PCE	Active area (cm^2)	Ref.
FTO/c-TiO_2/ mp-(TiO_2/ZrO_2/carbon) ($MAPbI_3$)	13.9	855	0.61	7.2%	0.07	1
FTO/c-TiO_2/ mp-(TiO_2/Al_2O_3/carbon) ($MAPbI_3$)	22.43	893	0.75	15.0%	0.09	2
FTO/c-TiO_2/ mp-(TiO_2/Al_2O_3/carbon) ($MAPbI_3(SrCl_2)_{0.1}$)	20.20	1050	0.75	15.9%	0.16	3
FTO/c-TiO_2/ mp-(TiO_2/ZrO_2/NiO/carbon) ($MAPbI_3$)	21.36	917	0.76	14.9%	NA	4
FTO/c-TiO_2/ mp-(TiO_2(Al_2O_3)/ZrO_2/carbon) ($MAPbI_3$)	22.89	950	0.62	13.5%	0.107	5
FTO/c-TiO_2/ mp-(TiO_2/ZrO_2/Co_3O_4/carbon) (MA(AVA-I)PbI_3)	23.43<br>23.11<br>22.10	880<br>950<br>915	0.64<br>0.53<br>0.57	13.3%<br>11.7%<br>11.4%	0.09<br>0.8<br>70	6
FTO/c-TiO_2/ mp-(TiO_2/Al_2O_3/carbon(WO_3)) ($MAPbI_3$)	17.28	933	0.63	10.8%	0.3	our work

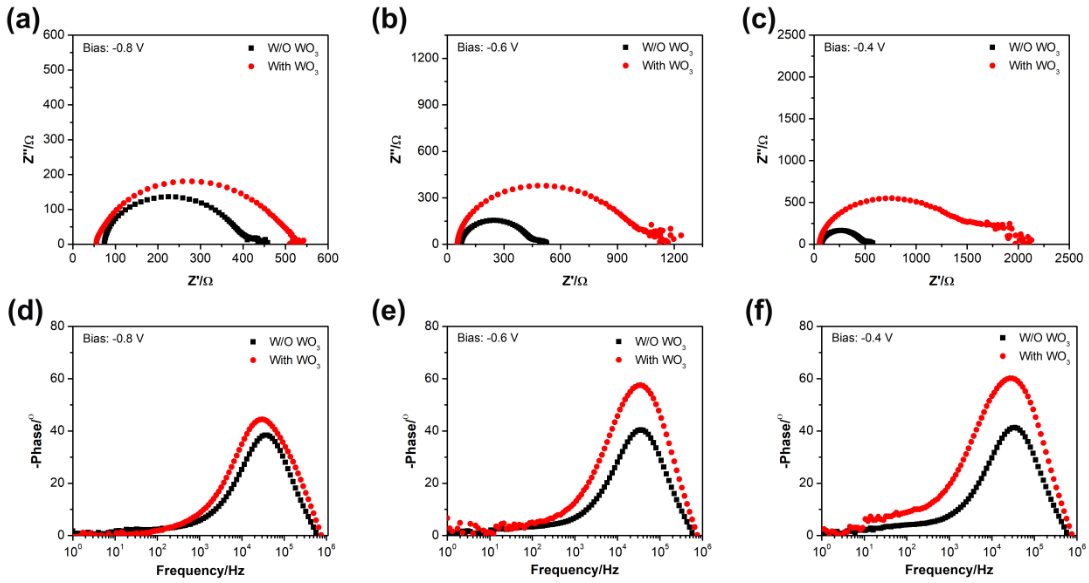

Figure S6. Electronic impedance spectroscopy (EIS) characteristics of PSCs with (With WO_3, red circle) or without (W/O WO_3, black square) WO_3 additive under no extra illumination. Electronic impedance spectrum in the form of Nyquist plots (up) and Bode phase plots (down) measured

under dark with bias at −0.8, -0.6 and -0.4 V.

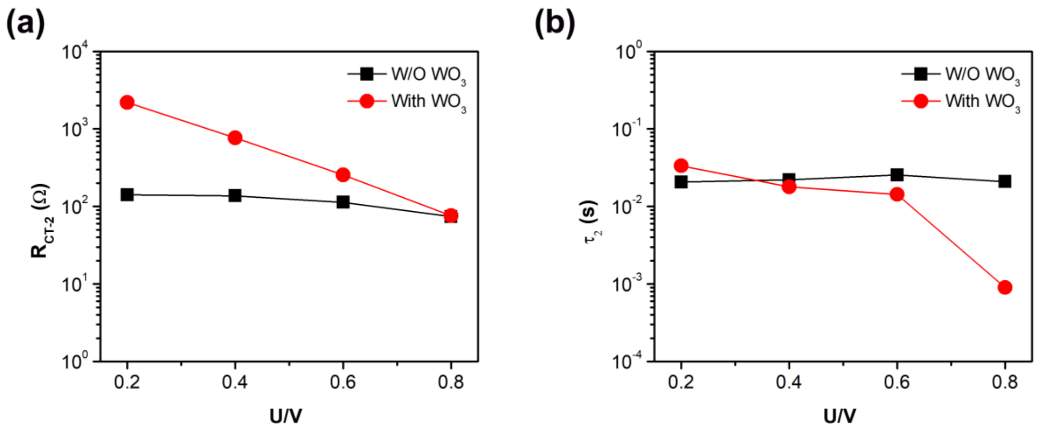

Figure S7. Plot of charge recombination resistance (R_{CT-2}) and lifetime constant (for τ_2) corresponding to the ion motion in perovskite in the PSCs without WO_3 (black square) and with WO_3 additive (red circle) obtained from impedance measurements under no extra illumination at the given bias.

Table S2. EIS spectra fitting data.

Applied Bias (V)	Sample	Rs (Ω)	R_{CE} (Ω)	R_{CT-1} (Ω)	R_{CT-2} (Ω)	C_{CE} (*10^{-8}F)	C_1 (*10^{-7}F)	C_2 (*10^{-5}F)	τ_1 (*10^{-5}s)	τ_2 (*10^{-2}s)
-0.2	W/O WO_3	75	141	249	141	3.61	1.46	14.6	3.64	2.06
	With WO_3	55	415	1426	2195	4.21	1.07	1.52	15.3	3.34
-0.4	W/O WO_3	75	136	232	137	3.39	1.5	16.1	3.48	2.21
	With WO_3	54	347	913	768	4.82	1.15	2.33	10.5	1.79
-0.6	W/O WO_3	75	120	217	113	3.02	1.3	22.5	2.82	2.54
	With WO_3	55	311	551	255	3.67	1.08	5.59	5.95	1.43
-0.8	W/O WO_3	76	109	189	74	2.88	1.31	28.3	2.48	2.09
	With WO_3	56	6	388	76	0.19	1.07	1.21	4.15	0.09

Table S3. Values for TRPL characteristics (Figure 5b) of perovskite on mp-Al_2O_3, mp-carbon and mp-carbon (WO_3) films.

Sample	τ_1 (ns)	Ratio1	τ_2 (ns)	Ratio2	$\tau_{ave}{}^{\#}$
Perovskite/Al_2O_3	0.51	57.6%	13.34	42.6%	5.98ns
Perovskite/Carbon	0.40	77.3%	11.37	22.7%	2.89ns
Perovskite/Carbon(WO_3)	0.38	84.6%	6.04	15.4%	1.25ns

$^{\#}\tau_{ave}$ = Ratio1*τ_1+ Ratio2*τ_2

1. Mei, A.; Li, X.; Liu, L.; Ku, Z.; Liu, T.; Rong, Y.; Xu, M.; Hu, M.; Chen, J.; Yang, Y.; Grätzel, M.; Han, H. A hole-conductor–free, fully printable mesoscopic perovskite solar cell with high stability. *Science* **2014,** *345* (6194), 295-298.

2. Tsai, C.-M.; Wu, G.-W.; Narra, S.; Chang, H.-M.; Mohanta, N.; Wu, H.-P.; Wang, C.-L.; Diau, E. W.-G. Control of preferred orientation with slow crystallization for carbon-based mesoscopic perovskite solar cells attaining efficiency 15%. *J. Mater. Chem. A* **2017,** *5* (2), 739-747.

3. Zhang, H.; Wang, H.; Williams, S. T.; Xiong, D.; Zhang, W.; Chueh, C. C.; Chen, W.; Jen, A. K. SrCl2 Derived Perovskite Facilitating a High Efficiency of 16% in Hole-Conductor-Free Fully Printable Mesoscopic Perovskite Solar Cells. *Adv. Mater.* **2017,** *29* (15).

4. Xu, X.; Liu, Z.; Zuo, Z.; Zhang, M.; Zhao, Z.; Shen, Y.; Zhou, H.; Chen, Q.; Yang, Y.; Wang, M. Hole selective NiO contact for efficient perovskite solar cells with carbon electrode. *Nano Lett.* **2015,** *15* (4), 2402-8.

5. Xiong, Y.; Zhu, X.; Mei, A.; Qin, F.; Liu, S.; Zhang, S.; Jiang, Y.; Zhou, Y.; Han, H. Bifunctional Al2O3 Interlayer Leads to Enhanced Open-Circuit Voltage for Hole-Conductor-Free Carbon-Based Perovskite Solar Cells. *Solar RRL* **2018,** *2* (5), 1800002.

6. Bashir, A.; Shukla, S.; Lew, J. H.; Shukla, S.; Bruno, A.; Gupta, D.; Baikie, T.; Patidar, R.; Akhter, Z.; Priyadarshi, A.; Mathews, N.; Mhaisalkar, S. G. Spinel Co3O4 nanomaterials for efficient and stable large area carbon-based printed perovskite solar cells. *Nanoscale* **2018,** *10* (5), 2341-2350.
